# Discovery of Shiga Toxin-Producing *Escherichia coli* (STEC)-Specific Bacteriophages From Non-fecal Composts Using Genomic Characterization

**DOI:** 10.3389/fmicb.2019.00627

**Published:** 2019-04-02

**Authors:** Yen-Te Liao, Xincheng Sun, Irwin A. Quintela, David F. Bridges, Fang Liu, Yujie Zhang, Alexandra Salvador, Vivian C. H. Wu

**Affiliations:** ^1^Produce Safety and Microbiology Research Unit, U.S. Department of Agriculture, Agricultural Research Service, Western Regional Research Center, Albany, CA, United States; ^2^Henan Key Laboratory of Cold Chain Food Quality and Safety Control, Zhengzhou University of Light Industry, Zhengzhou, China; ^3^Collaborative Innovation Center of Food Production and Safety, Zhengzhou, China; ^4^College of Food Science and Engineering, Ocean University of China, Qingdao, China; ^5^College of Food Science and Technology, Shanghai Ocean University, Shanghai, China

**Keywords:** Shiga toxin-producing *E. coli* (STEC), complementary host range, STEC-specific bacteriophages, whole genome sequencing, non-fecal composts

## Abstract

Composting is a complex biodegradable process that converts organic materials into nutrients to facilitate crop yields, and, if well managed, can render bactericidal effects. Majority of research focused on detection of enteric pathogens, such as Shiga toxin-producing *Escherichia coli* (STEC) in fecal composts. Recently, attention has been emphasized on bacteriophages, such as STEC-specific bacteriophages, associated with STEC from the fecal-contaminated environment because they are able to sustain adverse environmental condition during composting process. However, little is known regarding the isolation of STEC-specific bacteriophages in non-fecal composts. Thus, the objectives were to isolate and genomically characterize STEC-specific bacteriophages, and to evaluate its association with STEC in non-fecal composts. For bacteriophage isolation, the samples were enriched with non-pathogenic *E. coli* (3 strains) and STEC (14 strains), respectively. After purification, host range, plaque size, and phage morphology were examined. Furthermore, bacteriophage genomes were subjected to whole-genome sequencing using Illumina MiSeq and genomic analyses. Isolation of top six non-O157 and O157 STEC utilizing culture methods combined with PCR-based confirmation was also conducted. The results showed that various STEC-specific bacteriophages, including vB_EcoM-Ro111lw, vB_EcoM-Ro121lw, vB_EcoS-Ro145lw, and vB_EcoM-Ro157lw, with different but complementary host ranges were isolated. Genomic analysis showed the genome sizes varied from 42kb to 149kb, and most bacteriophages were unclassified at the genus level, except vB_EcoM-Ro111lw as FelixO1-like viruses. Prokka predicted less than 25% of the ORFs coded for known functions, including those essential for DNA replication, bacteriophage structure, and host cell lysis. Moreover, none of the bacteriophages harbored lysogenic genes or virulence genes, such as *stx* or *eae*. Additionally, the presence of these lytic bacteriophages was likely attributed to zero isolation of STEC and could also contribute to additional antimicrobial effects in composts, if the composting process was insufficient. Current findings indicate that various STEC-specific bacteriophages were found in the non-fecal composts. In addition, the genomic characterization provides in-depth information to complement the deficiency of biological features regarding lytic cycle of the new bacteriophages. Most importantly, these bacteriophages have great potential to control various serogroups of STEC.

## Introduction

In recent years, produce outbreaks associated with contamination of enteric pathogens, such as Shiga toxin-producing *Escherichia coli* (STEC), have drawn significant attention of health administrations and the public. During 2011, a severe outbreak in Europe was associated with contamination of fenugreek sprouts with *E. coli* O104:H4. There were approximately 3800 cases reported in Germany alone, including 845 cases of hemolytic uremic syndrome and 54 deaths (European Food Safety Authority, 2011; Frank et al., 2011; Beutin and Martin, 2012). Recently, a massive recall occurred in the United States regarding contamination of romaine lettuce with *E. coli* O157:H7 in 22 states, causing illness in 98 people, 46 hospitalizations, and 10 cases of hemolytic uremic syndrome (CDC, 2018). Produce is often served raw and, thus, is susceptible to any contamination from farm to fork. Contamination of pathogenic STEC strains on produce firstly occurs on the farm mostly derived from the contact with fecal-contaminated sources, such as wild animal, irrigation water and composts (Walters et al., 2011; Cooley et al., 2013).

Composting is a biodegradable process by microorganisms to turn organic materials to humus-like components that are commonly used as fertilizers or soil amendment to improve crop yields (Ryckeboer et al., 2003). An efficiently managed composting process would attribute to the reduced levels of pathogens, such as *Salmonella* and pathogenic *E. coli*, first present in the raw organic materials after the process due to the change of temperature and pH (Ryckeboer et al., 2003; Heringa et al., 2010). Inadequate composting processes have resulted in the spread of foodborne pathogens in pre-harvest environment (Buck et al., 2003), which are commonly associated with the composting of organic material with higher levels of pathogens, particularly of fecal origin (Handley et al., 2012). A previous study demonstrated that *Salmonella* and *E. coli* O157:H7 were able to survive through the niche of composting process and sustain at low levels in dairy-based compost (Kim et al., 2009). Another study further indicated that the use of *E. coli* O157:H7-polluted compost for soil amendment was directly associated with contamination of outer lettuce leaves during irrigation (Oliveira et al., 2012). Due to the natural reservoir of STEC, additional interventions have been used to minimize the pathogens in feces-based composts prior to use to prevent the spread of the pathogens (Lemunier et al., 2005; Heringa et al., 2010).

Bacteriophages (or phages) are key elements of intestinal microbiota of animals (Handley et al., 2012). Previous study indicated that various STEC-specific phages that were lytic against different serogroups of STEC strains, such as O26, O111 and O157, were isolated from cattle feces (Viazis et al., 2011). The authors found that these phages were free of virulence genes, such as *stx*, *hlyA*, and *eaeA* genes, and were potential biocontrol candidates to prevent STEC contamination. Another study, however, found that Stx phages – the phage containing *stx* genes – with lysogenic factors were present in the environment contaminated with animal feces and might be associated with emergence of new pathogens through the transfer of the virulence gene by the phages (Martinez-Castillo and Muniesa, 2014). Additionally, phages have been demonstrated to be able to be present at different stages of composting process (Johannessen et al., 2005; Partanen et al., 2010). The phages specific to *Pseudomonas* were isolated and characterized in composts (Amgarten et al., 2017); however, the information regarding the presence of STEC-specific phage or Stx phages in the composts, particularly non-fecal bases, is scarce. It is believed that the presence of STEC-specific phages or Stx phages (temperate phages) might have different impact on the biosafety level of compost upon application. Therefore, the objectives of this study were to isolate and genomically characterize free STEC-specific bacteriophages, and to evaluate its association with STEC strains in the non-fecal composts. To the best of the authors’ knowledge, this is the first study to isolate and characterize STEC-associated phages in non-fecal composts.

## Materials and Methods

### Sample Collection

Two non-fecal compost samples (C1 and C2) were randomly collected from a composting operation in California. The raw ingredients used for the composting were consisted of food scraps, from restaurants and markets, and yard trimmings.

### Isolation of STEC Strains

Using an abridged version of the protocol outlined in Cooley et al. (2013), samples were enriched and streaked on MacConkey Sorbitol Agar supplemented with cefixime and potassium tellurite (CT-SMAC), Rainbow Agar O157 supplemented with novobiocin and potassium tellurite (NT-RA), BBL Blood Agar supplemented with calcium chloride, mitomycin and X-gal (mSBA), and Chromagar O157. Typical colonies of STEC found on each type of media were picked and grown overnight in Luria Broth (LB) at 37°C and plated on Luria Agar. DNA from each isolated colony streaked from the two enriched samples was extracted and screened for *stx1*, *stx2abc*, *stx2f* and *stx2ex* using multiplex PCR (hot start at 95°C for 10 min, 38 cycles of 95°C for 20 s and 60°C for 45 s) with 2.5 units of AmpliTAQ gold, 1× supplied PCR buffer, 3 mM MgCl_2_, 200 mM dNTP, 300 nM of each primer, and 200 nM each probe to make a 20 μl reaction (Cooley et al., 2013). An *E. coli* O157:H7 (ATCC 35150) positive control and a *Salmonella* Typhimurium (ATCC 14028) negative control were included in each run. A Ct value of ≤ 28 for any of the four *stx* genes was considered to be a positive result.

### Preparation of Bacterial Host Strains for Phage Isolation

Fourteen STEC strains, including 2 strains per serogroups of O26, O45, O103, O111, O121, O145, and O157, from the culture collections of Produce Safety and Microbiology Research Unit in USDA ARS were used as host strains in this study (Supplementary Table S1). Non-pathogenic strains, including ATCC 13706, ATCC 43888, and DH5α, were used for the isolation of phages encoding *stx* genes (or Stx phages). Overnight cultures were prepared by inoculating each non-pathogenic and STEC strain in an individual tube with 5 ml of Tryptic Soy Broth (TSB, Difco, Becton Dickinson, Sparks, MD, United States) and incubated overnight at 37°C. The cocktails of non-pathogenic and STEC strains were prepared separately by adding 0.1 ml of each overnight culture from each group together shortly prior to use.

### Isolation of Free Bacteriophages

Two non-fecal composts in 50 ml conical tubes were gently mixed by hand prior to centrifugation at 8,000 × *g* for 10 min to get rid of sediments. The supernatants were filtered through 0.22 μm syringe filter membrane, to get rid of background flora, and were used for phage isolation. In brief, an aliquot of 5 ml filtered supernatant from each sample was mixed with 25 ml TSB supplemented with 10 mM CaCl_2_ and inoculated with 0.3 ml of respective STEC and non-pathogenic *E. coli* cocktails for incubation at 37°C for 48 h. Next, chloroform (4% v/v) was added to the enriched samples and homogenized on a rotator (Thermo Fisher Scientific, Waltham, MA, United States) for 30 min to inactivate the residual host strains and background flora. The supernatants were obtained after centrifugation at 8,000 × *g* for 10 min and used to determine the presence of the phages against top six non-O157 STEC and O157 STEC strains by spotting 10 μl supernatants on Tryptic Soy Agar (TSA, Difco, Becton Dickinson, Sparks, MD, United States) containing one of the host strains (spot assay). After overnight incubation at 37°C, the plates with clearing on the spotted area, which indicated positive results, were further subjected to phage purification process as previously described (Liao et al., 2018). During purification process, two plaques with different plaque sizes were picked from the first single-layer agar plaque assay plates, and three runs of plaque-picking and enrichment process were conducted (Liao et al., 2018). After purification, each phage lysate was enriched in 40 ml TSB with the corresponding host culture at 37°C overnight for phage propagation. The propagated phages were filtered through 0.22 μm syringe filter membrane prior to further analysis.

### Determination of Host Range and Antimicrobial Effects Using Efficiency of Plating for the Isolated Phages

To determine host range and antimicrobial effects of the isolated phages, the spot assay was used as described above to spot the lysate of the isolated phages on the TSA plates pre-mixed with individual strain of the selected host panel, the same group as those used for phage isolation, and the level of the clearing (opaque, transparent and complete clearing) on the spotted area was used to determine the lysis ability of the phages against different serogroups of STEC. Efficiency of plating (EOP) was used to evaluate the lytic activity of the phages against a variety of bacterial strains. In brief, fresh overnight cultures of the tested bacterial strains and the primary bacterial strain used for phage isolation were prepared in TSB at 37°C. After serial dilution, the diluted phage solutions (10^-3^ to 10^-7^) were used to obtain titers with each strain using a double-layer agar plaque assay, and the plates were incubated at 37°C overnight. EOP was calculated based on phage titer obtained from the tested bacteria versus the phage titer from the primary bacterium used for phage isolation. If EOP was 0.5 or more, it was considered as high production efficiency. An EOP of 0.1 or above, but below 0.5, was considered as medium production efficiency. Low production efficiency had EOP between 0.001 and 0.1, and any value equal to or under 0.001 represented inefficiency of phage production.

### Determination of Plaque Morphology of the Isolated Phages

A stereo microscope (Stemi 305 cam, Carl Zeiss Microscopy, Germany) was used to observe plaque morphology from the double-layer agar plaque assay plates. The images of plaques were captured by use of Zeiss Labscope Application (Version 2.5.1).

### Concentration of Phages for Downstream Analysis

The filtered lysates were concentrated using Amicon Ultra-15 Centrifugal Filter Unit (Merck Millipore, Billerica, MA, United States) with 100 KDA cut-off. An aliquot of 10 ml phage lysate was added to Amicon filter unit, which was subsequently centrifuged using a fixed angle rotor at 5,000 × *g* according to manufacturer’s instruction. The filter unit was then washed with addition of 200 μl sterile H_2_O and centrifuged at 5,000 × *g* to get rid of the water, followed by adding 200 μl of 10 μg/ml DNase I and incubating at 37°C for 30 min to eliminate bacterial DNA contamination. After spinning down the DNase I, the filter was then washed with 5 ml of sterile H_2_O with the procedure as previously described. Subsequently, an aliquot of 500 μl of sterile H_2_O was used to re-suspend the phages retained on the filter membrane, and the phage solution was transferred to a sterile microcentrifuge tube. Twenty microliters of the concentrated phages were spared for electron microscopy observation, while the rest of the lysates were used for DNA extraction.

### Transmission Electron Microscopy

Each Amicon Filter-concentrated phage lysate with volume of 6 μl was placed on copper mesh PLECO grids (Ted Pella Inc., Redding, CA, United States) and incubated for 1 min at room temperature, followed by blotting on Whatman filter paper and subsequent negative staining with addition of 8 μl of 0.75% uranyl acetate (Sigma-Aldrich, Darmstadt, Germany) for incubation at room temperature (around 26°C) for 30 s. The phage morphology was then examined using a transmission electron microscope (Tecnai G2 F20 model FEI, United States).

### Detection of *stx* Genes in the Phages

The concentrated phage lysates were then subjected to DNA extraction using a phage DNA extraction kit (Norgen Biotek Corp., ON, Canada). Presence of *stx1* and *stx2* genes in the phages was screened by PCR as previously described (Liao et al., 2018).

### Whole Genome Sequencing and Genomic Analyses

Prior to library construction, the purity and concentration of each phage’s DNA was determined by electrophoresis on 1% agarose gel and Nanodrop 8000, respectively. Two hundred nanograms of DNA were fragmented to 550 bp by Ultrasonic Cell Disruptor (M220 Covaris) and subsequently subjected to DNA library construction using a TruSeq^®^ Nano DNA Library Prep Kit (Illumina, San Diego, CA, United States) according to manufacturer’s instruction. The libraries were sequenced on Illumina MiSeq sequencer using a MiSeq reagent kit v3 (Illumina, 600 cycles) with approximately 2 million paired-end (250-bp) sequence reads. The sequenced reads were subjected to FastQC, and the raw sequences were then trimmed using Trimmomatic on Galaxy online server^1^. A *de novo* assembly of the resultant quality reads was conducted using Unicycler v0.4.1 (SPAdes) with default parameters on Galaxy. The assembled sequences were subjected to NCBI (National Center for Biotechnology Information) BLAST to obtain taxonomic classification, including order, family, and genus. The phage termini and possible packaging mechanisms were predicted according to the *in silico* determination method proposed in PhageTerm (Garneau et al., 2017). The final contigs were annotated with Prokaryotic genome annotation (Prokka, v.1.12.0) with default parameters (Seemann, 2014). The open reading frames (ORFs) predicted and annotated by Prokka were further subjected to BLASTN search to confirm the annotation results. The reference phage genomes used in this study, including *Escherichia* phage ECML-117 (GenBank accession number JX128258.1), *Escherichia* phage ESCO13 (GenBank accession number KX552041.2), *Escherichia* phage K1ind1 (GU196279.1), and *E. coli* O157 typing phage 12 (GenBank accession number KP869110.1) were obtained based on the maximum score of the published sequences on NCBI nucleotide database after the BLASTN search using the assembled sequences of the new phages from this study. A visualized circular map with the predicted ORFs for each of vB_EcoM-Ro111lw, vB_EcoM-Ro121lw, vB_EcoS-Ro145lw, and vB_EcoM-Ro157lw genomes were constructed using Geneious v11.0.4 (Biomatters Ltd., Auckland, New Zealand). The comparative analyses of phage nucleotide sequences, between the newly isolated phages and the corresponding reference phages, were conducted using EasyFig (Sullivan et al., 2011). ClustalW algorithm v2.1 (McWilliam et al., 2013) and the neighbor-joining method was used to evaluate evolutionary relationship of the phages based on the genes of interest (Saitou and Nei, 1987). The evolutionary distances were computed using the Maximum Composite Likelihood method (Tamura et al., 2004) and were in the units of the number of base substitutions per site. The analysis involved 4 nucleotide sequences. All positions containing gaps and missing data were eliminated. There was a total of 955 positions in the final dataset. Evolutionary analyses were conducted in MEGA7 (Kumar et al., 2016). The whole-genome sequences of *Escherichia* phages vB_EcoM-Ro111lw, vB_EcoM-Ro121lw, vB_EcoS-Ro145lw, and vB_EcoM-Ro157lw were deposited to NCBI GenBank under accession number MH571750, MH160766, MH051334, and MH051335, respectively.

## Results and Discussion

### Isolation of STEC Strains and STEC-Specific Phages From the Composts

For STEC isolation, a total of 17 and 23 presumptive positive colonies were picked from selective media after incubation of streak-plated enriched C1 and C2 samples, respectively. None of the colonies was positive of *stx* genes by PRC screening (Table 1). The results without isolation of STEC strains were possibly due to non-fecal materials of the compost samples used in this study.

**Table 1:** Isolation of STEC strains and STEC-specific bacteriophages from non-fecal composts.

Sample	Isolation of STEC strains	Isolation of STEC-specific phages^a^
C1	none	O111, O121, O145, O157
C2	none	O111. O121, O145, O157, ATCC 43888, ATCC 13706, and DH5α


*^*a*^Results were determined by spot assay against different serogroups of STEC and non-pathogenic strains.*

**FIGURE 1:**
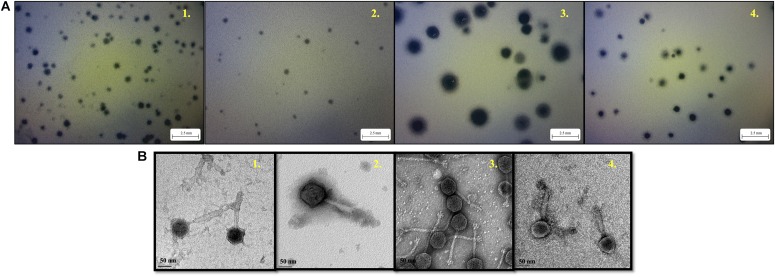
The morphology of plaques formed by phage vB_EcoM-Ro111lw **(A1)**, phage vB_EcoM-Ro121lw **(A2)**, phage vB_EcoS-Ro145lw **(A3),** and phage vB_EcoM-Ro157lw **(A4)** on their host strains of STEC O111, O121, O145, and O157, respectively, using plaque assay, and the transmission electron microscopy images of phage vB_EcoM-Ro111lw **(B1)**, phage vB_EcoM-Ro121lw **(B2)**, phage vB_EcoS-Ro145lw **(B3)**, and phage vB_EcoM-Ro157lw **(B4)**.

With regard to phage isolation, the spot assay results showed that both C1 and C2 contained phages lytic against the same serogroups of STEC strains, including O111, O121, O145, and O157, regardless of lysis levels on the plates. Additionally, the phages against non-pathogenic *E. coli* strains, including DH5α, ATCC 13706, and ATCC 43888 (*E. coli* O157:H7 without *stx* genes), were also detected by spot assay in the C2 (Table 1). The non-pathogenic hosts were used primarily for the isolation of Stx phages from the samples; however, after phage purification, none of these phages, including both STEC-specific phages and those lytic against non-pathogenic strains, were positive of *stx* genes, indicating that no Stx phages were isolated from the non-fecal composts (data not shown). Based on degree of lysis from spot assay, four phages, one of each against serogroup of O111, O121, O145, and O157 strains, were selected for further biological and genomic analyses. The four phages, including *Escherichia* phages vB_EcoM-Ro111lw, vB_EcoM-Ro121lw, vB_EcoS-Ro145lw, and vB_EcoM-Ro157lw, were named after the phage morphology, the species name and serogroup of the major bacterial host accordingly (Adriaenssens and Brister, 2017). The TEM observation showed that vB_EcoS-Ro145lw belonged to the family *Siphoviridae*, while the other 3 phages were *Myoviridae* (Figure 1B). Furthermore, vB_EcoS-Ro145lw produced the largest size of plaques among all phages, indicating its strong lytic effect against its host strain, STEC O145 (Figure 1A). The host range test indicated that, in addition to generic *E*. *coli*, such as ATCC 13706 and DH5α strains, both phages vB_EcoM-Ro111lw and vB_EcoM-Ro121lw were lytic (antimicrobial) against multiple serogroups of STEC strains, whereas vB_EcoS-Ro145lw and vB_EcoM-Ro157lw had narrow host ranges (Table 2). In addition, EOP results revealed that vB_EcoM-Ro111lw was the only phage showing medium or high infection efficiency against different serogroups (O26 and O111) of STEC strains.

**Table 2:** Host range and antimicrobial effects of the isolated bacteriophages against various STEC and non-pathogenic strains by spot assay^α^ and EOP^β^ (efficiency of plating).

*E. coli* strains	Phage vB_EcoM-Ro111lw		Phage vB_EcoM-Ro121lw		Phage vB_EcoS-Ro145lw		Phage vB_EcoM-Ro157lw	
				
	Spot assay	EOP	Spot assay	EOP	Spot assay	EOP	Spot assay	EOP
O26-1	++	0.03	-	NT	-	NT	-	NT
O26-2	++	0.03	-	NT	-	NT	-	NT
O45-1	-	NT	+	<0.001	-	NT	-	NT
O45-2	+	<0.001	+ + +	<0.001	-	NT	-	NT
O103-1	+	<0.001	-	NT	-	NT	-	NT
O103-2	+	<0.001	-	NT	-	NT	-	NT
O111-1	+ + +	H^ϕ^	-	NT	-	NT	-	NT
O111-2	+ + +	0.4	-	NT	-	NT	-	NT
O121-1	-	NT	+ + +	0.7	-	NT	-	NT
O121-2	-	NT	+ + +	H^ϕ^	-	NT	-	NT
O145-1	-	NT	+	<0.001	+ + +	H^ϕ^	-	NT
O145-2	-	NT	+	<0.001	+ + +	0.9	-	NT
O157-1	-	NT	+	<0.001	-	NT	+ + +	H^ϕ^
O157-2	+	<0.001	+	<0.001	-	NT	+ + +	0.9
ATCC 43888^∗^	+	<0.001	+	<0.001	-	NT	+ + +	0.8
DH5α^∗^	+	<0.001	-	NT	-	NT	++	<0.001
ATCC 13706^∗^	++	<0.001	+	0.1	+	<0.001	++	<0.001


*^α^Spot assay is used to determine the host range of the phages; Degree of lysis is indicated using cross mark, “+” indicates weak lysis, “++” indicates medium lysis, “+ + +” indicates complete lysis and “–” indicates no lysis. ^β^EOP is conducted only for the spot assay-positive strains; NT indicates EOP was not tested; EOP = phage titer on the test strain/ phage titer on the primary strain used for phage isolation; EOP equal (=) or above (>) 0.5 means high production efficiency, 0.5 > EOP ≥ 0.1 means medium production efficiency, 0.1 > EOP > 0.001 means low production efficiency, and EOP equal (=) or bellow (<) 0.001 means inefficiency of phage production. ^ϕ^The primary strain used for phage isolation. ^∗^Non-pathogenic *E. coli* strains include ATCC 43888, DH5α and ATCC 13706.*

A complete composting process is capable of reducing a significant amount of pathogens present in the raw organic materials (Erickson et al., 2009). However, if the composting organic materials containing high level of enteric pathogens, such fecal matters, was inefficiently managed, the final compost product may pose a risk of the spread of pathogens on a farm (Wu et al., 2009). Phages have been demonstrated to be more resistant to the environmental stress, such as the drastic change of pH and temperature, than bacteria and, thus, are likely to sustain through the composting process (Johannessen et al., 2005). Current findings revealed that various STEC-specific phages were isolated from the composting of non-fecal materials, such as yard trimmings or food scraps. Although the initial levels of pathogens in the raw materials prior to composting were unknown, the presence of STEC-specific phages was likely to add additional antimicrobial factors to reduce STEC to non-detectable levels during the process. Shiga toxin-producing *E. coli* (STEC)-specific phages were usually isolated from feces or fecal-contaminated environment (Imamovic et al., 2010; Wang et al., 2015; Amarillas et al., 2016); however, current study reported the presence of STEC-specific phages and evaluated its correlation with the STEC bacterial hosts from non-fecal composts.

### Genomic Features of Four STEC-Specific Phages

The genome sizes of phages vB_EcoM-Ro111lw, vB_EcoM-Ro121lw, vB_EcoS-Ro145lw, and vB_EcoM-Ro157lw were 86,950bp, 149,803bp, 42,145bp, and 72,179bp, respectively (Table 3). According to taxonomy report of BLASTN search at NCBI nucleotide database, the phages vB_EcoM-Ro111lw, vB_EcoM-Ro121lw, and vB_EcoM-Ro157lw belonged to the family *Myoviridae* and the vB_EcoS-Ro145lw belonged to *Siphoviridae*; the taxonomic results were in consensus to the morphologies observed under TEM of these phages. Additionally, the phage vB_EcoM-Ro111lw was further classified under sub-family *Ounavirinae* as FelixO1-like viruses; however, the rest of 3 phages were unclassified at genus level. The three myophages isolated in this study had genome sizes ranging from 72 k to 149 k bp in which the results were in agreement with the finding of the previous study indicating a wide range of genome sizes of the phages belonging to *Myoviridae* were isolated from cattle feces (Wang et al., 2015). The GC content of phage vB_EcoS-Ro145lw (50.6%) was not only higher than that of other new phages, with 46, 39.1, and 38.8% for vB_EcoM-Ro157lw, vB_EcoM-Ro121lw, and vB_EcoM-Ro111lw, respectively, but also close to that of *E. coli* strains with an average GC content of 50%. A previous study found that lytic phages usually had an average GC content 4% lower than that of its bacterial host, whereas temperate phages (with both lytic and lysogenic cycles) had GC content very close to the host (Rocha and Danchin, 2002). Although the GC content of phage vB_EcoS-Ro145lw was similar to that of its O145 STEC host strain, the phage expressed strong lysis, such as forming big plaques against the strain, without having any sign of lysogenic factor. On the other hand, the GC contents of both phages vB_EcoM-Ro121lw and vB_EcoM-Ro111lw were significant lower than that of their *E. coli* hosts (∼50.7%), suggesting the host strains, O121 and O111 STEC, used for the phage isolation in this study were not the optimal hosts for these two phages (Bahir et al., 2009). The small plaque sizes formed by phages vB_EcoM-Ro121lw and vB_EcoM-Ro111lw against its original strains of isolation –O121 and O111 STEC strain, respectively – might correlate with these findings.

**Table 3:** Genomic features of the STEC-specific bacteriophages from non-fecal composts.

Features	vB_EcoM-Ro111lw	vB_EcoM-Ro121lw	vB_EcoS-Ro145lw	vB_EcoM-Ro157lw
Taxonomic classification at genus level	FelixO1-like phage	Unclassified phage	Unclassified phage	Unclassified phage
Accession number	MH571750	MH160766	MH051334	MH051335
Genome size (bp)	86,950	149,803	42,145	72,179
GC content (%)	38.8	39.1	50.6	46
No. of ORFs predicted	153	292	71	109
No. of ORFs code for known proteins	28	44	17	15
No. of ORFs code for hypothetical proteins	103	237	54	94
No. of ORF code for tRNAs	22	11	0	0


### Prediction of Genome Termini and Packaging Mechanisms

The phage termini and possible packaging mechanism of the isolated phages were predicted according to the bioinformatic algorithm proposed in PhageTerm (Garneau et al., 2017). The analytical results indicated that both phages vB_EcoS-Ro145lw and vB_EcoM-Ro121lw had circularly permuted direct terminal repeats (without cohesive ends) with the packing mechanism similar to the well-known phage P1: headful with *pac* site (Oliveira et al., 2013). In the headful packaging mode, the terminase would recognize and cleave at a unique sequence region, called *pac*, to generate the first termini and initiate the packaging of the genomic DNA into the prohead (Li et al., 2014). The packing mechanisms for both phages vB_EcoM-Ro111lw and vB_EcoM-Ro157lw were predicted to be direct terminal repeats (DTR) at the genome termini without circular permutation: vB_EcoM-Ro111lw had a 613-bp terminal repeat region (similar to the phage T7, short direct terminal repeats) and vB_EcoM-Ro157lw had a 3533-bp terminal repeat region at both ends of the genome (similar to the phage T5, long direct terminal repeats) (Casjens and Gilcrease, 2009). Generally, in the mode of terminal repeats, the terminal sequences were generated by duplicating the direct repeat DNA sequence together with packaging; however, the mechanism regarding the generation of long repeats was unknown (Casjens and Gilcrease, 2009). Although the principles of several well-known packaging mechanisms were elucidated, identification of these packaging mechanisms from newly isolated phages traditionally require generation of restriction maps that are usually complicated with results not easy to interpret (Li et al., 2014). With the benefits of the computation of whole genome sequencing data using PhageTerm software, it was able to easily predict the terminal ends and types of the packaging mechanisms of these new phages (Garneau et al., 2017).

### Genome Analysis of Four STEC-Specific Phages

**FIGURE 2:**
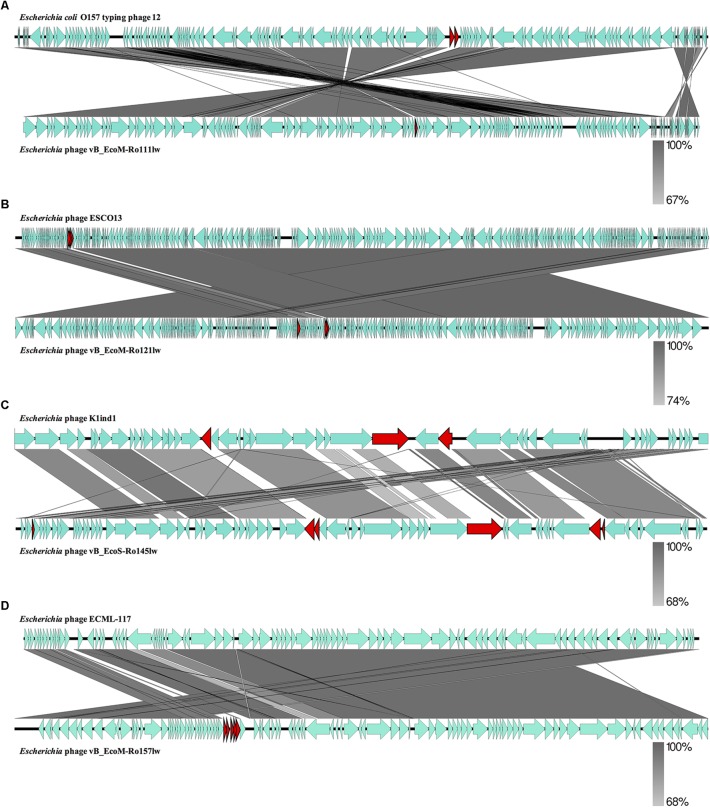
Whole genome comparison of *Escherichia* phage vB_EcoM-Ro111lw **(A)**, *Escherichia* phage vB_EcoM-Ro121lw **(B)**, *Escherichia* phage vB_EcoS-Ro145lw **(C)**, and *Escherichia* phage vB_EcoM-Ro157lw **(D)** with their reference genomes from NCBI database using EasyFig. A gray-scaled shaded area indicates the different levels of sequence similarity between two phage sequences. The arrow shapes indicate the annotated ORFs in the genome, and the unshared ORFs are highlighted as red arrows.

The BLASTN searches of the new phage genomes in this study against the reference sequences on NCBI database varied among different phages. The two best hits for vB_EcoS-Ro145lw genome included *Escherichia* phage P AB-2017 (67% coverage and 90% identity) and *Escherichia* phage K1ind1 (70% coverage and 89% identity); the low genome coverage of the new phages against its reference genomes indicated the difference of the phage as opposed to the known genomes among *Siphoviridae* phages from the public database. The BLASTN search results also showed that vB_EcoM-Ro111lw, vB_EcoM-Ro121lw, and vB_EcoM-Ro157lw had the best hits against *E. coli* O157 typing phage 12, 11, and 8 (all with 95% coverage and 98% identity), *Escherichia* phage ESCO13 (98% coverage and 99% identity), and *Escherichia* phage ECML-117 (94% coverage and 97% identity), respectively. Although the phages vB_EcoM-Ro111lw, vB_EcoM-Ro121lw, and vB_EcoM-Ro157lw had higher sequence coverage of genetic similarity than that of vB_EcoS-Ro145lw against their reference sequences, vB_EcoM-Ro111lw was the only phage further classified at genus level, FelixO1-like phages, based on NCBI taxonomy report. However, the phage vB_EcoM-Ro111lw was unable to infect *Salmonella*, unlike the *Salmonella* phage Felix O1, the core phage in the genus, with capability against different serovars of *Salmonella* strains (Whichard et al., 2010). BLAST comparison of the newly isolated phages and their reference phage genomes using EasyFig showed that that the genomes of vB_EcoM-Ro111lw and *E. coli* O157 typing phage 12 shared approximately over 90% nucleotide sequence similarity. However, one annotated ORF, coding for hypothetical protein, in phage vB_EcoM-Ro111lw was absent in the genome of *E. coli* O157 typing phage 12; two ORFs from the reference genome were lacking in vB_EcoM-Ro111lw (Figure 2A). The results also showed a high degree of nucleotide sequence similarity (> 90%) between the phages vB_EcoM-Ro121lw its reference genome – *Escherichia* phage ESCO1 – with 3 unshared ORFs, primarily coding for hypothetical proteins, from each other (Figure 2B). For vB_EcoS-Ro145lw genome, the results showed that low degree of nucleotide sequence similarity (68%) was observed on some shared ORFs as compared to the phage K1ind1 (Figure 2C). Additionally, there were 6 ORFs in vB_EcoS-Ro145lw that were absent in the counterpart genome and 3 ORFs from the reference genome that were not shared in the genome of vB_EcoS-Ro145lw. The comparison between the phage vB_EcoM-Ro157lw and *Escherichia* phage ECML-117 indicated a high nucleotide sequence similarity between the two genomes and all the annotated ORFs from the reference phage genome were shared in the vB_EcoM-Ro157lw; five ORFs in vB_EcoM-Ro157lw that coded for hypothetical proteins were absent in the reference phage (Figure 2D). These results showed that the genomes of the new phages and their counterparts contained some sequence regions with ORFs that were identified with the predicted hypothetical proteins in high similarity but in different organization in the genomes. The phenomenon, also known as mosaicism, likely deriving from horizontal genetic transfer may account for the diversity of phage genomes (Hatfull and Hendrix, 2011).

**FIGURE 3:**
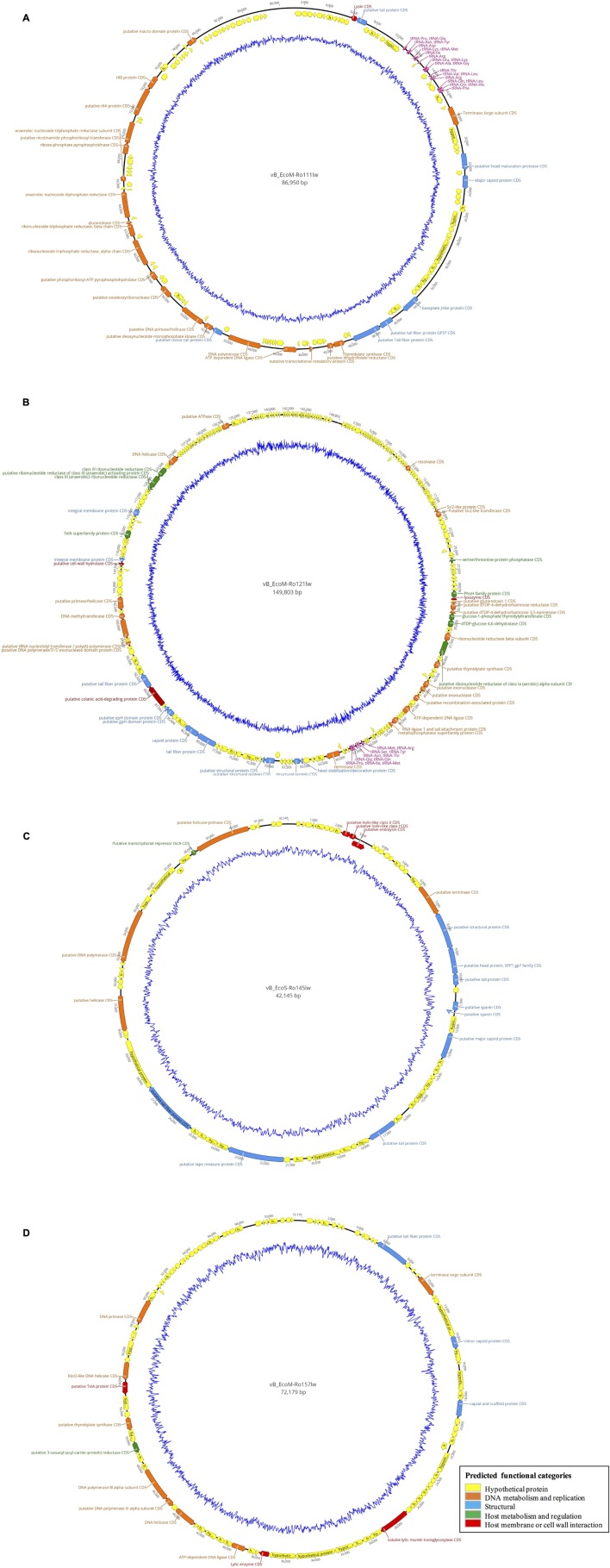
Circular genome map of the phages vB_EcoM-Ro111lw **(A)**, vB_EcoM-Ro121lw **(B)**, vB_EcoS-Ro145lw **(C)**, and vB_EcoM-Ro157lw **(D)**. The outer circle represents genes as indicated by the arrows. Predicted functional categories of the genes were determined according to annotation and are represented with different colors. The central graph in blue shows genomic GC content variation.

According to genome annotation, the predicted number of ORFs for each of phage vB_EcoM-Ro111lw, vB_EcoM-Ro121lw, vB_EcoS-Ro145lw, and vB_EcoM-Ro157lw were 153, 292, 71, and 109, respectively (Table 3). There were more than 75% of the ORFs annotated with unknown function, even including the FelixO1-like phage vB_EcoM-Ro111lw. The phage genomes that contained the ORFs with the predicted functions essential for phage structure, DNA metabolism and replication host lysis proteins, and host metabolism and regulation were illustrated with different colors in Figure 3. Additionally, only vB_EcoM-Ro111lw and vB_EcoM-Ro121lw contained tRNAs, with 22 and 10, respectively. Five of the tRNAs, #2, #10, #18, #20, and #21, in the vB_EcoM-Ro111lw genome and one, #4, in vB_EcoM-Ro121lw genome may be pseudo-tRNAs (Supplementary Table S2). Most tRNAs in these two phage genomes were located in the region adjacent to the DNA packing cluster, which might be associated with earlier stage of phage progenies formation (Bailly-Bechet et al., 2007). Bailly-Bechet et al. (2007) also indicated that lytic phages would contain more tRNAs, if present, than lysogenic phages. Moreover, it was demonstrated that the presence of tRNAs was more likely associated with the fitness of the phages, such as ability to infect more hosts, in order to adapt to different environment (Delesalle et al., 2016). Their findings were in consensus to the results of wide host ranges for the phages vB_EcoM-Ro111lw and vB_EcoM-Ro121lw in this study. In the genome of vB_EcoM-Ro157lw, the ORF #41 was predicted with the function of soluble lytic murein transglycosylase, which had the similar function as lysozyme/endolysin to catalyze the cell wall of its bacterial hosts, causing cell lysis (van Asselt et al., 1999), while the ORF #12, ORF #81 and ORF #11 were predicted as lysozyme/endolysin in the vB_EcoM-Ro111lw, vB_EcoM-Ro121lw, and vB_EcoS-Ro145lw genomes, respectively (Supplementary Table S3–S6). Furthermore, in vB_EcoM-Ro111lw genome, two ORFs were annotated as putative dihydrofolate reductase (#77) and thymidylate synthase (#76) that were found to be the unique molecular markers for Bastille-like phages, a newly proposed genus of myophages in sub-family *Spounavirinae*, which were also known for the wide host ranges (Asare et al., 2015). The phage vB_EcoM-Ro111lw was classified as Felix O1-like phages according to NCBI taxonomy report, even though it was predicted to harbor the ORFs coded these two key markers, dihydrofolate reductase and thymidylate synthase, from other genus, Bastille-like phages, which demonstrated the evolutionary perspective of the phages likely through horizontal genetic transfer in the environment. A previous study found that a *Salmonella-*specific phage, UAB_Phi20, with detection of lysogenic genes still showed strong lytic infection against its *Salmonella* hosts; however, the lack of lysogenic cycle was derived from the suppression of the phage’s packaging mechanism, resulting in the dominant lytic cycle of the phage (Bardina et al., 2016). Though expression of strong lytic cycle, the presence of lysogenic genes in the phages may not be appropriate for the potential biocontrol use. In the current study, the genomic analysis showed that no lysogenic or virulence genes, such as *stx* or *eae* genes, were detected in the genomes of these four newly isolated phages.

### Comparative Genome Analysis

**FIGURE 4:**
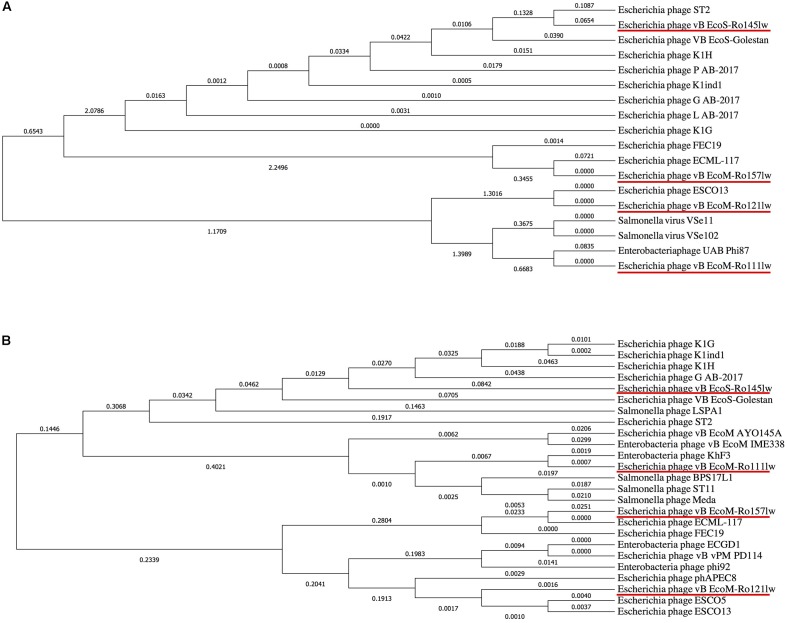
Phylogenetic comparison based on Clustal Omega alignment of the genes coding for tail fiber **(A)** and endolysin/lysozyme **(B)** from the newly isolated phages (red underline) and other reference phage genomes obtained from NCBI database. The tree is drawn to scale, with branch lengths (next to the branches) in the same units as those of the evolutionary distances used to infer the phylogenetic tree.

It was hard to create a cluster of genes based on the sequences in this study because of the lack of similarity of these phage gnomes. Therefore, the ORFs encoded the proteins responsible for the function of host specificity and lytic effects, such as tail and lysozyme, were selected to construct phylogenetic analyses for a better understanding of the evolution of tailed phages with regard to its host range (Haggård-Ljungquist et al., 1992). The phylogenetic analysis based on ORFs encoding tail fiber showed both phages vB_EcoM-Ro111lw and vB_EcoM-Ro121lw were in the clade, indicating a closer evolutionary relationship with regard to the tail fiber (Figure 4A). The phages vB_EcoS-Ro145lw and vB_EcoM-Ro157lw were also in the same clade, which were closely related to *Escherichia* phage ST2 and phage ECML-117, respectively (Figure 4A). The results were in consensus to the previous biological features in which both phages vB_EcoM-Ro111lw and vB_EcoM-Ro121lw had a wider host range. With regard to endolysin/ lysozyme, the phylogenetic comparison indicated that phages vB_EcoM-Ro121lw and vB_EcoM-Ro157lw were in the same clade: the phage vB_EcoM-Ro157lw was closely related to phage ECML-117 whereas phage vB_EcoM-Ro121lw was further clustered into a single branch (Figure 4B). Similar results were also observed on the phages vB_EcoS-Ro145lw and vB_EcoM-Ro111lw that phage vB_EcoM-Ro111lw was closely related to phage KhF3 and phage vB_EcoS-Ro145lw was clustered into a single branch (Figure 4B).

## Conclusion

In conclusion, the present study provides valuable information with regard to the isolation of various STEC-specific phages and the correlation of these phages with the STEC bacterial hosts from non-fecal compost samples. Whole-genome sequencing enabled us to comprehensively characterize the newly isolated phages, together with biological features, to determine further applications of the phages. Furthermore, genomic analyses of these newly isolated phages provide in-depth information regarding the evolution and diversity of STEC-specific phages isolated from non-fecal environment. The phages isolated in this study do not contain lysogenic factors potentially associated with the transfer of unwanted genes among bacterial population. The four new phages, including vB_EcoM-Ro111lw, vB_EcoM-Ro121lw, vB_EcoS-Ro145lw, and vB_EcoM-Ro157lw, together are able to infect a wide range of STEC strains, including serogroups of O157 and top 6 non-O157, due to their different but complementary host ranges. Without the presence of virulence genes, such as *stx* or *eae* genes and lysogenic factors, these phages are potential candidates used to control different serogroups of STEC strains. Moreover, evidence of the phenotypic and genotypic characteristics associated with the mitigating effects of these phages may account for zero isolation of STEC strains in this study. These findings suggest that the lytic phages are of great potential in controlling various STEC strains and may provide additional antimicrobial effects against STEC that are present in the raw composting materials and sneaked through the composting process if not well-managed. Further studies regarding the reduction of STEC by these phages under various conditions are necessary in order to optimize the antimicrobial activities against STEC in different food-associated environments.

## Data Availability

The datasets generated for this study can be found in Genbank, MH571750, MH160766, MH051334, and MH051335.

## Author Contributions

Y-TL was responsible for phage isolation with XS, data analyses, genome annotation, and manuscript preparation. FL and YZ were responsible for phage sequencing. DB was responsible for STEC strain isolation. IQ and AS were responsible for TEM and host range test. VW conceived the study, aided in experiment design, and prepared the manuscript. All authors reviewed the manuscript.

## Conflict of Interest Statement

The authors declare that the research was conducted in the absence of any commercial or financial relationships that could be construed as a potential conflict of interest.

## Footnotes

1 https://usegalaxy.org
